# Association of serum cortisol and dehydroepiandrosterone sulfate (DHEAS) levels with psychological stress in patients with vitiligo

**DOI:** 10.3906/sag-1812-84

**Published:** 2019-06-18

**Authors:** Ahmet GÜRPINAR, Sibel DOĞAN GÜNAYDIN, Cengiz KILIÇ, Ayşen KARADUMAN

**Affiliations:** 1 Department of Dermatology and Venereology, Faculty of Medicine, Hacettepe University, Ankara Turkey; 2 Department of Psychiatry, Faculty of Medicine, Hacettepe University, Ankara Turkey

**Keywords:** Vitiligo, stress, cortisol, dehydroepiandrosterone sulfate

## Abstract

**Background/aim:**

Vitiligo is a depigmentation disorder that leads to serious psychological burden in patients, who are frequently reported to have depression and anxiety. The aim of this study was to investigate the association between stress-related hormone levels and psychological stress in vitiligo.

**Materials and methods:**

In this study 46 vitiligo patients and 46 controls were enrolled; their cortisol, dehydroepiandrosterone sulfate (DHEAS), and cortisol/DHEAS levels were measured. Psychological burden was assessed by the Beck Depression Inventory and Perceived Stress Scale.

**Results:**

Patients and controls did not differ in terms of cortisol, DHEAS, or cortisol/DHEAS. Patients had higher perceived stress than controls but did not differ in terms of depression scores. Correlation analyses revealed that cortisol/DHEAS correlated positively with perceived stress (P = 0.009, r = 0.272). The correlation between cortisol/DHEAS and perceived stress was stronger in the patient group (P = 0.013, r = 0.363) and close to zero among controls. In regression analyses, lower depression and higher perceived stress were shown to predict cortisol/DHEAS values.

**Conclusion:**

Vitiligo patients significantly differed from the healthy population in terms of hormones and psychological distress. There was also an association between perceived stress and cortisol/DHEAS ratio in vitiligo. Abnormality of hormonal response to distress lowers DHEAS, which is known for its antioxidant properties, a possible mechanism for vitiligo development. Another important finding is the significance of using the composite variable cortisol/DHEAS, which seems to be more sensitive to distress than each of its components. We suggest its use in future studies on psychological distress–hormone relationships.

## 1. Introduction 

Stress and hormones are known to be closely related, and recent studies show that, in addition to cortisol, the major stress hormone dehydroepiandrosterone sulfate (DHEAS) may also play a role in stress [1,2]. Psychogenic stress is also thought to be an important factor in vitiligo, which is an acquired pigmentation disorder characterized by circumscribed depigmented macules and patches on the skin. One of the most popular pathogenetic mechanisms suggested for the pathophysiology of vitiligo is oxidative stress and hydrogen peroxide accumulation, leading to damage in melanocytes in the epidermis [3,4]. The cosmetic disfigurement is known to lead to serious psychological burdens in patients with vitiligo, who are frequently reported to have depression and anxiety disorders, which may in part be caused by stigmatization of the disease [5–7]. It is also possible that psychological distress or depression, through their effects on stress hormones, may have a role in the development of vitiligo. 

Based on the existing literature, we sought to investigate the association between stress-related hormone levels (serum cortisol and DHEAS) and psychological stress measures in vitiligo patients. To our knowledge, DHEAS levels were not evaluated in vitiligo before. The relationship between hormone levels and clinical subtypes of vitiligo was also evaluated. Our hypotheses were as follows:

1. We will find differences in stress-related hormone levels (i.e. cortisol and DHEAS) between vitiligo patients and controls. 

2. We will find higher psychological distress and depression in vitiligo patients than controls.

3. Psychological distress and depression will be related to hormone levels in vitiligo patients, but not in controls.

## 2. Materials and methods

### 2.1. Sample and study design

#### 2.1.1. Samples 

Vitiligo patients who had been admitted to dermatology outpatient clinic of the Hacettepe University Hospital in 2010 and 2011, who were older than 18 years of age, and who had given consent for participation were included in the study. Sex- and age-matched controls were chosen from among healthy individuals. 

#### 2.1.2. Assessments 

Exclusion criteria were defined as the coexistence of adrenal, pituitary, and psychiatric diseases or polycystic ovary syndrome (PCOS). In order to exclude a probable diagnosis of PCOS, females with abnormally high free testosterone levels, irregular menstrual periods, and hirsutism were excluded. All participants who had abnormally high free testosterone and DHEAS levels, any systemic chronic disease, systemic infection, pregnancy, or a history of medication use including systemic steroids, oral contraceptives, and psychiatric drugs within 3 months prior to the study were excluded. 

#### 2.1.3. Procedure 

All participants were asked to avoid heavy exercise, cigarette smoking, and alcohol intake prior to the day of blood sampling. Peripheral venous blood samples were taken from patients and controls in the morning between 0800 and 0900 hours after a 12-h fasting period. Morning cortisol, DHEAS levels, and cortisol/DHEAS ratios were analyzed at the Hacettepe University Hospital Central Laboratories by chemiluminescence method using the IMMULITE 2000 assay. Psychological distress was assessed by the Beck Depression Inventory (a 21-item self-report measuring the intensity of depressive symptoms) and the Perceived Stress Scale (a 10-item psychological instrument for measuring perceptions of stress, designed to measure how unpredictable, uncontrollable, and overloaded respondents find their lives) [8,9]. Generalized vitiligo was defined as vitiligo with acrofacial lesions (distal fingers and periorificial areas) and/or widely distributed patches or with a mixed pattern of both involving more than 10% of the body surface area (BSA). Focal vitiligo was defined as one or more macules in only one body area or dermatome and with less than 10% BSA involvement. Ethical approval was given by the Hacettepe University Ethics Board (LUT 12/04-22). 

### 2.2. Statistical analysis

Outcome measures were mean DHEAS and cortisol values. A composite variable that is commonly used in the literature, the cortisol/DHEAS ratio, was also used as an outcome variable. In order to test our hypotheses, we compared hormone levels between patient and control groups using t-tests. Correlation analyses were carried out between hormone levels, psychological distress, and perceived stress scale scores. Predictors of hormone levels were assessed using linear regression analyses. SPSS 17.0 was used in the statistical analyses.

## 3. Results

A total of 46 vitiligo patients (27 males and 19 females) and 46 sex- and age-matched controls were enrolled in the study. Serum cortisol, DHEAS, and cortisol/DHEAS levels in the patient and control groups are shown in Table 1. Contrary to our first hypothesis, the patient and control groups did not differ in terms of morning cortisol, DHEAS levels, or cortisol/DHEAS ratios. 

**Table:** Comparison of study group and controls according to hormone levels and psychological stress.

	Patients, n = 46(mean ± SD)	Controls, n = 46(mean ± SD)	P
Cortisol	14.05 ± 5.03	13.78 ± 6.24	0.446
DHEAS	192.31 ± 106.07	224.82 ± 109.15	0.158
Cortisol/DHEAS	0.12 ± 0.14	0.08 ± 0.06	0.113
Perceived Stress Scale	18.43 ± 6.24	15.96 ± 5.02	0.097
Beck Depression Inventory	10.46 ± 8.87	7.33 ± 7.53	0.095

Our second hypothesis was partly supported; the patient group reported higher perceived stress than controls, although the two groups did not differ in terms of depression scores. 

To test our third hypothesis, we ran correlation analyses in the total group first and then we repeated the same analyses within patients and controls separately (Table 2). In the total sample (n = 92), cortisol/DHEAS correlated positively with perceived stress (DHEAS tended to relate negatively to psychological distress). Cortisol levels did not show any correlations with distress or depression. 

**Table 2:** Correlations between hormone levels and psychological stress in patient and control groups.

Subjects		Cortisol		DHEAS		Cortisol/DHEAS	
Patient group	Perceived Stress Scale	P	0.406	P	0.219	P	0.013
		r	0.126	r	–0.185	r	0.363
	Beck Depression Inventory	P	0.154	P	0.930	P	0.179
		r	0.216	r	0.13	r	0.040
Control group	Perceived Stress Scale	P	0.054	P	0.351	P	0.735
		r	–0.286	r	–0.141	r	–0.051
	Beck Depression Inventory	P	0.070	P	0.248	P	0.964
		r	–0.27	r	–0.174	r	–0.07
Total	Perceived Stress Scale	P	0.499	P	0.069	P	0.009
		r	–0.71	r	–0.19	r	0.272
	Beck Depression Inventory	P	0.696	P	0.342	P	0.654
		r	–0.042	r	–0.101	r	0.048

r: Pearson correlation coefficient.

When examined separately, the correlation between cortisol/DHEAS and perceived stress was stronger in the patient group and close to zero among controls (Figure). This suggests that the patient group was responsible for the correlation observed.

**Figure:**
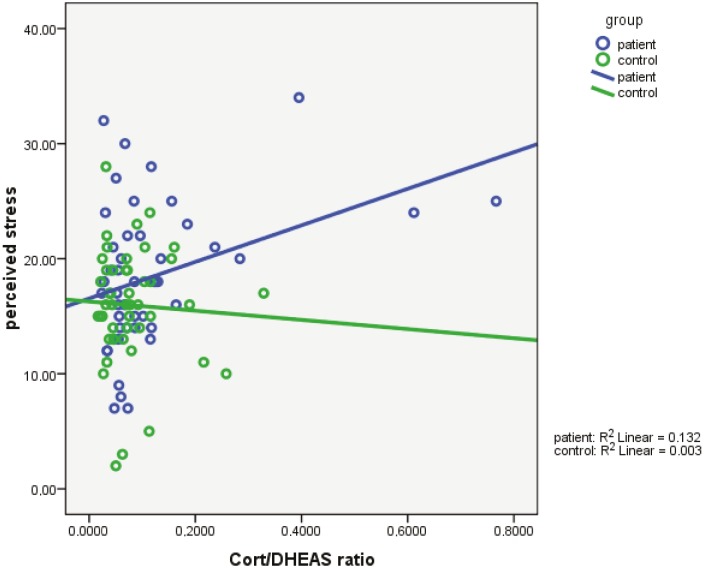
Correlation between cortisol/DHEAS and perceived stress in vitiligo patients and controls.

To further test our third hypothesis, this time controlling for the intervening variables, we ran regression analyses using cortisol, DHEAS, and cortisol/DHEAS as dependent variables in turn. We wanted to see if group membership (belonging to patient vs. control group) predicted hormone levels, and if this prediction was independent of sex, age, or psychological distress. We therefore entered the predictor (explanatory) variables in three steps: group membership first, sex and age second, and distress measures (depression and perceived stress) in the last step (Table 3). The predictors of DHEAS and cortisol/DHEAS were understandably similar: both were predicted by sex and age. DHEAS levels were predicted by younger age and being male, whereas cortisol/DHEAS ratio was predicted by older age and being female. Cortisol levels were predicted by being female, and there was a trend for younger age. DHEAS was also predicted by belonging to the control group, and there was a trend for the opposite for cortisol/DHEAS. While neither cortisol nor DHEAS were found related to psychological distress measures, cortisol/DHEAS was predicted by both: lower depression and higher perceived stress predicted cortisol/DHEAS values. 

**Table 3:** Predictors of hormone levels in total group, patients, and controls.

Predictor variables	Cortisol		DHEAS		Cortisol/DHEAS	
Total group	β	P	β	P	β	P
Group (1 = patients, 2 = controls)	–0.060	0.571	0.151	0.042	–0.151	0.100
Sex (1 = males, 2 = females)	0.263	0.018	–0.472	0.000	0.406	0.000
Age	–0.173	0.098	–0.598	0.000	0.274	0.003
Depression	–0.014	0.925	–0.001	0.988	–0.250	0.046
Perceived distress	–0.153	0.309	0.018	0.865	0.272	0.037
Patients	β	P	β	P	β	P
Sex (1 = males, 2 = females)	–0.166	0.369	–0.611	0.000	0.316	0.041
Age	–0.178	0.257	–0.584	0.000	0.325	0.014
Depression	0.202	0.365	0.113	0.418	–0.349	0.060
Perceived distress	0.058	0.810	0.026	0.862	0.465	0.023
Controls	β	P	β	P	β	P
Sex (1 = males, 2 = female)	0.445	0.002	–0.389	0.001	0.555	0.000
Age	–0.096	0.474	–0.601	0.000	0.256	0.051
Depression	–0.179	0.301	–0.095	0.513	–0.007	0.965
Perceived distress	–0.166	0.343	0.057	0.698	–0.118	0.481

We repeated the above analyses within patient and control groups separately (Table 3), to see if there was a differential prediction of hormone levels between groups. The prediction of DHEAS and cortisol/DHEAS by age and sex was similar to those of the total sample. The prediction of cortisol/DHEAS by distress measures, however, was limited only to the patient group: the prediction was stronger in the patient group and was close to zero among controls.

Finally, we examined the patient group to see if illness-related variables such as extent of lesions, family history, and illness duration had any effect on hormone levels. Cortisol was not predicted by any of the variables. Both DHEAS and cortisol/DHEAS were predicted by age and sex. The only variable that was predicted by clinical variables was DHEAS; shorter illness duration and more generalized lesions predicted higher DHEAS levels (Table 4). 

**Table 4:** Predictors of hormone levels in the patient group including clinical variables.

Predictor variables	Cortisol		DHEAS		Cortisol/DHEAS		β	P	β	P	β	P
	Sex (1 = males, 2 = females)	0.241	0.146	–0.470	0.000	0.401	0.008
Age (years)	–0.256	0.131	–0.549	0.000	0.259	0.087
Illness duration (years)	0.088	0.637	–0.257	0.043	0.082	0.618
Body area affected (1 = <10%, 2 = ≥10%)	–0.294	0.160	0.281	0.045	–0.111	0.544
Family history of vitiligo (0 = no, 1 = yes)	–0.048	0.757	0.144	0.168	–0.104	0.455
Perceived distress (0–40)	0.023	0.927	–0.147	0.371	0.361	0.105
Depression (0–63)	–0.159	0.518	0.055	0.736	–0.277	0.206

## 4. Discussion 

The present study is the first to examine stress hormone levels and their relationship to psychological distress in vitiligo patients. Patients with vitiligo are frequently reported to have depression and anxiety disorders, which may in part be caused by stigmatization of the disease [5–7]. However; to our knowledge, stress hormone levels and their possible interactions in vitiligo have not been studied in this patient population in the literature before. Although the stress hormone levels did not significantly differ between patients and controls, the regression analyses taking into account other intervening variables showed that the two groups actually differed in terms of DHEAS levels; the control group had higher DHEAS levels. Since the patient group reported higher psychological distress than the control group, controlling for distress (and depression) was warranted. Consequently, we found that DHEAS levels in the control group were independent of distress levels. However, this did not mean that distress was independent of hormone levels. In within-group analyses, perceived stress was related to cortisol/DHEAS in the patient group, but not in the control group. Although we showed that vitiligo patients had lower DHEAS than controls and were highly distressed, these points alone cannot show that vitiligo is caused by distress or low DHEAS, or both. Still, we believe it is important that the stress and cortisol/DHEAS relationship was observed in patients and not in controls, which could mean that hormonal response to distress may be abnormally elevated in some people, a possible mechanism for the development of vitiligo. A further, although weak, support for this speculation was observed in analyses looking into disease severity: we observed a tendency for those who had a longer illness duration to have lower DHEAS levels. DHEAS is known for its antioxidant properties; it is therefore reasonable to assume that lack of DHEAS may be a reason for longer duration of illness, or lack of adequate response to treatment [8–11]. 

One important finding of this study has to do with the significance of using the composite variable of cortisol/DHEAS. This ratio seems to be more sensitive to distress than each of its components. We suggest its use in future studies on psychological distress–hormone relationships. 

Another interesting finding, which looks counterintuitive, is about the relationship between DHEAS levels and extent of the lesions. It seems that the more generalized the lesions, the higher the DHEAS levels. This relationship is independent of disease duration (which is longer in the generalized type) and distress or depression levels (those with generalized lesions tended to have lower depression scores). Our definition of the extent of vitiligo lesions was a dichotomous variable. It is possible that we may have missed some correlations that could have been found if we had measured the extent of lesions using a finer (continuous) measure. 

Our study has several limitations. The collected data were cross-sectional. It was therefore not possible to reach a definitive conclusion on causality. Low sample size did not allow us to include more variables in regression analyses; studies with larger samples are warranted.

In conclusion, the present study is the first to examine the relationship between cortisol, DHEAS, and psychological distress in vitiligo patients. Our results provide some insight into the interaction between stress hormones and psychological distress in the development of vitiligo. Our conclusion is that hormonal response to distress may be abnormally elevated in some people, a possible mechanism for the development of vitiligo. Studies with larger samples are needed to clarify the nature of the proposed interaction.

## Acknowledgement

The Hacettepe University Scientific Investigations Unit funded this study.
